# Epidrugs: novel epigenetic regulators that open a new window for targeting osteoblast differentiation

**DOI:** 10.1186/s13287-020-01966-3

**Published:** 2020-10-28

**Authors:** Mahsa Ghorbaninejad, Maliheh Khademi-Shirvan, Samaneh Hosseini, Mohamadreza Baghaban Eslaminejad

**Affiliations:** 1 Basic and Molecular Epidemiology of Gastrointestinal Disorders Research Center, Research Institute for Gastroenterology and Liver Diseases, Shahid Beheshti University of Medical Sciences, Tehran, Iran; 2 Department of Stem Cells and Developmental Biology, Cell Science Research Center, Royan Institute for Stem Cell Biology and Technology, ACECR, Tehran, Iran; 3 Department of Genetics, Reproductive Biomedicine Research Center, Royan Institute for Reproductive Biomedicine, ACECR, Tehran, Iran; 4 Department of Cell Engineering, Cell Science Research Center, Royan Institute for Stem Cell Biology and Technology, ACECR, Tehran, Iran

**Keywords:** Epigenetic, Epidrug, Mesenchymal stem cell, Osteoblast

## Abstract

Efficient osteogenic differentiation of mesenchymal stem cells (MSCs) is a critical step in the treatment of bone defects and skeletal disorders, which present challenges for cell-based therapy and regenerative medicine. Thus, it is necessary to understand the regulatory agents involved in osteogenesis. Epigenetic mechanisms are considered to be the primary mediators that regulate gene expression during MSC differentiation. In recent years, epigenetic enzyme inhibitors have been used as epidrugs in cancer therapy. A number of studies mentioned the role of epigenetic inhibitors in the regulation of gene expression patterns related to osteogenic differentiation. This review attempts to provide an overview of the key regulatory agents of osteogenesis: transcription factors, signaling pathways, and, especially, epigenetic mechanisms. In addition, we propose to introduce epigenetic enzyme inhibitors (epidrugs) and their applications as future therapeutic approaches for bone defect regeneration.

## Background

Mesenchymal stem cells (MSCs) are considered to be potent tools for regenerative medicine, tissue engineering, and cell-based therapy because of their tri-lineage differentiation, self-renewal potential, low immunogenicity, decreased risk for tumorigenicity, capability for expansion, and ease of accessibility [1]. During bone homeostasis and bone fracture repair, MSCs differentiate into osteoblasts that ultimately result in bone formation and regeneration. However, the application of MSCs in cell-based therapy may present challenges, such as unexpected differentiation of MSCs in different in vivo situations and low survival of MSCs after transplantation. Thus, it is necessary to have a better understanding of the osteogenesis process and its exact regulatory mechanism [2, 3]. The process of osteogenic differentiation of MSCs is largely controlled by successive changes in the pattern of gene expression related to osteogenesis. Osteoblast differentiation of MSCs is highly modulated by cross-talk between genes, transcription factors, signaling pathways, and epigenetic mechanisms [4]. Epigenetic modulators alter chromatin architecture and change accessibility of genes to transcription factors and other regulators. These changes are largely responsible for the up- and downregulation of specific genes during a cell’s lifespan [5]. Disruption of epigenetic regulation is associated with human diseases, including those responsible for abnormalities in development, cancer, and neuropsychiatric disorders. Hence, an epigenetic study of the gene expressions involved in the osteogenic differentiation pathway would be beneficial in order to understand the process of osteogenesis. Major epigenetic mechanisms include DNA methylation, histone modifications, chromatin remodeling, and miRNA [6]. One attractive strategy that can target epigenetic change is the application of chemical modifiers as epigenetic enzyme inhibitors, termed epidrugs. These epidrugs may represent a step forward in cancer therapy and treatment of other diseases in which epigenetic regulation plays a key role [7]. The effects of epidrugs have been confirmed in relevant biological models. Several chemical modifiers have been studied in the induction of epigenetic regulation in MSC differentiation toward the osteoblast lineage [8]. In this article, we review the genes, transcription factors, signaling pathways, and, particularly, epigenetic mechanisms that govern the osteogenic process. We discuss the role of epigenetic changes and their modifiers as one of the most promising and expanding fields in MSC differentiation. Finally, the impact of epidrugs as attractive candidates for the regulation of epigenetic mechanisms in osteoblast differentiation of MSCs is discussed.

## Regulation of osteogenesis by genes and signaling pathways

Osteoblastogenesis has three important phases that are characterized by sequential expression of specific osteoblastic markers: proliferation, matrix maturation, and mineralization. Major genes involved in the osteoblast differentiation process include alkaline phosphatase (ALP), type 1 collagen (COL1A1), osteopontin (OPN), bone sialoprotein (BSP), and osteocalcin (OCN) [9]. ALP, BSP, and COL1A1 are typically expressed during the early stage of osteoblast differentiation, while OCN is expressed during the late stage and is associated with mineralization. Runt-related transcription factor 2 (Runx2) and osterix (Osx) are two important transcription factors that promote osteoblastogenesis [10].

Runx2, the most important transcription factor, is essential for bone formation and activation of osteogenic genes [11]. Runx2 acts as a master regulator upstream of the genes and transcription factors that are involved in osteogenesis. It binds to a specific site of the OCN, COL1A1, ALP, and OPN gene promoters [12]. Runx2 also regulates Osx expression, another major transcription factor for osteogenic differentiation [13]. Similarly, Osx, known as Sp7, is expressed by osteoblasts. Osx is essential for bone formation. Osx activates COL1A1 by interaction with its promoter [14, 15].

In addition to specific genes in the osteogenesis process, there are several known signaling pathways that play critical roles during osteoblast differentiation. MSCs proliferate and differentiate in response to these signaling pathways during different stages of osteogenesis. Activation of specific genes mediated by molecular signaling pathways regulates MSC differentiation [16]. Hedgehog (Hh), Notch, Wnt, parathyroid hormone (PTH), fibroblast growth factor (FGF), and the transforming growth factor-β (TGF-β) super family signaling are the most well-known signaling pathway regulators of osteogenesis.

**Fig. 1:**
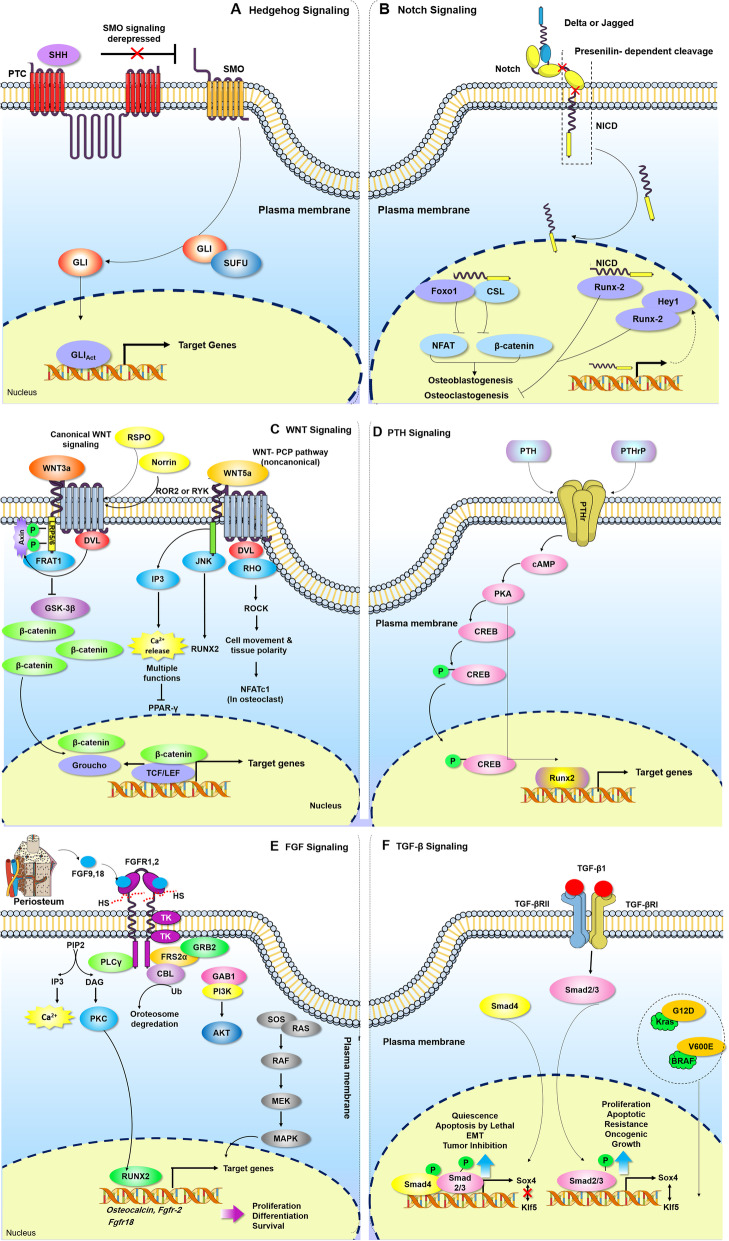
Osteogenesis regulating signaling pathways. **a** Hedgehog signaling pathway: Hh, an important secreted molecule of the hedgehog family, regulates cell functions during bone formation. Hh, hedgehog; Shh, Sonic hedgehog; Ihh, Indian hedgehog, Dhh; Desert hedgehog; Ptc, patched; Smo, smoothened. **b** Notch signaling pathway: Notch signaling has a key role in cellular development and tissue morphogenesis. Runx2, runt-related transcription factor 2; ADAM, a disintegrin and metalloprotease; TACE, tumor necrosis factor-α converting enzyme; NICD, Notch intracellular domain; CSL, C protein binding factor 1/suppressor of Hairless/Lag-1; MAML, mastermind-like, HES, hairy and enhancer of split; HEY, HES-related with YRPW motif. **c** Wnt signaling pathway: Wnt signaling is another signaling pathway in development and skeletal pattern. The noncanonical signaling pathway plays a role in regulating the osteoblast lineage. FZD, frizzled; LRP5 or LRP6, lipoprotein receptor-related protein 5 or 6; GSK3, glycogen synthase kinase 3; JNK, c-Jun N-terminal kinase. **d** PTH/ PTHrP signaling pathway: PTH may have either catabolic or anabolic effects on bone formation, depending on its route of administration. PTH, parathyroid hormone; PTHrP, PTH-related peptide; PTHR, PTH/PTHrP receptor; PKA, protein kinase A; CREB, cAMP response element binding protein. **e** FGF signaling pathway: FGF signaling plays an important role during skeletal development and it controls endochondral and intramembranous ossification. FGF, fibroblast growth factor. **f** TGF-β/BMP signaling: TGF-β and BMP signaling are of utmost importance in both bone formation during skeletal development and maintenance of postnatal bone. TGF-β, transforming growth factor-β; BMPs, bone morphogenetic proteins; R-Smad, receptor-regulated Smad; co-Smad, common-mediator Smad

Hh, an important secreted molecule of the hedgehog family, regulates cell functions during bone formation. The Hh receptor is expressed on the surface of osteoblasts. Shh, Ihh, and Dhh are members of the Hh family in mammals, and Shh and Ihh are vital for bone development. Family members of the Hh protein bind to its receptor, Ptc, which inhibits Smo, a key transducer in Hh signaling, and leads to activation of the GLI family (Gli1-3) transcription factors. Gli translocate into the nucleus and activate a series of Hh target genes (Fig. 1a). Results of studies based on rodent mesenchymal cell lines have demonstrated that Hh increased the expression of osteoblastic markers and mineralization [17].

In addition, Notch signaling has a key role in cellular development and tissue morphogenesis. Notch signaling requires cell-cell interaction between Notch receptors and Delta or Jagged family ligands present on the surface of the neighboring cells. Notch receptors activate by binding with ligands, which results in cleavage by ADAM/TACE metalloprotease, followed by cleavage with γ-secretase. This leads to release and nuclear translocation of NICD, where NICD interacts with the CSL family transcription factor and MAML co-activator. This complex targets HES and the HEY family of transcription factors, which control the expressions of other genes such as Runx2 (Fig. 1b). Conflicting results have been reported in terms of Notch signaling and regulation of osteoblast differentiation. Activation of Notch during the early stages of osteogenesis suppresses differentiation of osteoblasts from osteoprogenitors, while activation in the late stages exerts anabolic effects and causes excessive production of osteocytes from osteoblasts, resulting in bone formation [18]. Wnt signaling is another signaling pathway in development and skeletal pattern. Canonical Wnt signaling is activated by the Wnt1/3a protein when it binds to the G protein-coupled receptor and co-receptors at the cell surface, named FZD, and the low-density LRP5 or LRP6 complex. In the absence of Wnt, β-catenin is phosphorylated by a complex that contains GSK3. The ligand-receptor complexes inhibit GSK3, which prevents β-catenin degradation. β-catenin accumulates in the cytosol and translocates in the nucleus to regulate target gene transcription. In the Wnt-PCP pathway, Wnt binding to FZD activates Rho/Rack GTPase and the JNK signaling cascade (Fig. 1c). Wnt proteins regulate a wide range of cellular activities, including growth, cell fate determination, polarity, differentiation, migration, and proliferation [19].

Binding of PTH and PTHrP to PTHR leads to activation of the cAMP/protein kinase A (PKA) signaling pathway. Subsequently, PKA phosphorylates CREB, which regulates transcription of PTH target genes during osteogenesis (Fig. 1d). PTH may have either catabolic or anabolic effects on bone formation, depending on its route of administration. Continuous exposure of PTH results in bone resorption by activation of osteoclasts [20], whereas intermittent subcutaneous infusions lead to bone augmentation [21]. FGF signaling plays an important role during skeletal development, and it controls endochondral and intramembranous ossification [22]. Following ligand-receptor binding, intrinsic tyrosine residues of FGFR undergo dimerization and phosphorylation and, in turn, activate several signaling pathways such as MAP/ERK, PLCγ/PKCα, and PI3K-AKT (Fig. 1e) [22]. According to the literature, FGF2 and FGF18 are the most important regulators in promoting osteogenic differentiation, and they can enhance stabilization and expression of Runx2 [23].

The TGF-β superfamily consists of over 40 members, including TGF-βs, Activins, Nodal, bone morphogenetic proteins (BMPs), and growth differentiation factors (GDFs). TGF-β and BMP signaling are of utmost importance in both bone formation during skeletal development and maintenance of postnatal bone [24]. BMP family members are secreted by most skeletal cell types such as osteoprogenitor cells, osteoblasts, and bone extracellular matrix [25]. TGF-β is a multifunctional growth factor that has a complex role in bone remodeling. Although three isoforms of TGF-β (β1–β3) are synthesized by the majority of cells, platelets, endothelial cells, bone, and cartilage are the main sources of TGF-β [26]. In the Smad-dependent signaling pathway, BMP/TGF-β bind to their specific type II receptor, which recruits and phosphorylates a type I receptor. The type I receptor then phosphorylates and activates R-Smads. Smad1, Smad5, and Smad8 are BMP activated, whereas Smad 2 and 3 are TGF-β activated. R-Smads form a complex with co-Smad (Smad 4) and enter the nucleus to regulate transcription of target genes. BMPs can also transduce signals to Smad-independent signaling pathways, notably via MAP kinases (Fig. 1f). Of note, all of the mentioned signaling pathways interact with each other to modulate osteogenesis [27–31].

The interplay of molecular signals and genes enables MSCs to begin the differentiation process. The role of epigenetic modification in precise control of specific transcriptional programs and signaling pathways in the osteogenesis process has been less explored.

## Regulation of osteogenic differentiation by epigenetic mechanisms

Epigenetics are heritable changes in the patterns of gene expression that do not directly alter DNA nucleotide sequences. Epigenetic marks include DNA methylation, histone modifications, microRNA (miRNA), and chromatin remodeling in diverse biological processes. The results from a growing number of studies have indicated that these mechanisms participate in all aspects of osteogenesis [32]. Below, we provide a detailed description of epigenetic marks and their impact on osteogenesis.

### DNA methylation

DNA methylation is an epigenetic process that causes changes to chromatin that are associated with gene repression. In DNA methylation, a methyl group is added to the 5′ position of cytosine in a CpG dinucleotide [33]. Methylation is mediated by a number of DNA methyl transferases (DNMT) that consist of maintenance enzyme DNMT1 and de novo methyl transferases DNMT3A/3B. Methylation of DNA can be removed by demethylases, which include GADD45A, the TET family, and OCT4 [34].

DNA methylation associated with transcriptional silencing participates in biological processes such as imprinting of specific genes, X chromosome inactivation, and cell type-specific gene expressions [35]. According to numerous researches, DNA methylation plays a key role in osteogenesis of MSCs [36]. Delgado-Calle et al. have shown that an alteration in ALP gene expression was regulated by DNA methylation of its promoter region during osteogenesis in such a way that hypomethylation of the ALP promoter region in osteoblasts resulted in upregulation of the ALP gene. In contrast, reduced ALP expression in mature osteocytes resulted from hypermethylation of its promoter [37, 38]. Sepulveda et al. reported that DNA methylation processes control Sp7 gene expression during osteogenesis. They found that Sp7 gene silencing was mediated by DNMT1/3a. In contrast, activation of the Sp7 gene accompanied by DNA demethylation was mediated by SWI/SNF- and Tet1/Tet2-containing complexes [39]. Wakitani et al. reported that DNA methylation in a new genomic region was related to Runx2 expression; methylation of Runx2-DMR decreased during osteogenic differentiation in mice and canines [40]. Hypomethylation in promoters of other specific osteogenic genes, such as ROR2, Dlx5, Runx2, OCN, and Osx, occurs during osteogenic differentiation and results in increased mRNA expression [41, 42]. A recent study mentioned the pivotal role of DNA demethylases in the maintenance of MSCs and bone homeostasis. The results indicated that demethylases TET1 and TET2, through demethylation of the P2rX7 promoter and exosome releasing control, promote Runx2 signaling such that deletion of Tet1 and Tet2 in a mouse model led to an osteoporotic phenotype and impaired BMMSC differentiation [43]. These findings indicate that DNA methylation regulates gene expression during osteogenesis in a time-dependent manner.

### Histone modifications

Histone modifications are covalent post-translational modifications on the histone proteins. These modifications change the architecture of chromatin, which generally occurs at chemically unstable residues (i.e., lysine, arginine, serine, threonine, tyrosine, and histidine) on the histone tail regions. Histone tails are modified by acetylation, methylation, ubiquitination, phosphorylation, sumoylation, and ADP-ribosylation [44, 45]. Among these, most studies have focused on the role of histone acetylation and methylation during osteogenesis.

Histone acetylation is mediated by two classes of enzymes, histone acetyltransferases (HATs) and histone deacetylases (HDACs). Acetylation of lysine residues on histones neutralizes the partial electric charge of lysine, which relaxes the chromatin structure that correlates with transcriptional activation. In contrast, histone deacetylation compacts the chromatin structure and induces transcriptional repression [46].

Many studies have reported that histone acetylation regulates expression of osteo-specific genes. According to Shen et al., H3 and H4 acetylation was associated with fluctuations of OCN gene expression during osteogenesis. In the proliferative phase, acetylation of H3 and H4 decreased, which resulted in inactivation of OCN. In contrast, OCN was active in mature osteoblasts with increasing H4 acetylation [4]. Subsequently, Tan et al. performed ChIP-on-chip analysis on the full human genome promoter to study the role of histone modification during osteogenic differentiation of MSCs. Their findings revealed that H3K9Ac decreased while H3K9Me2 increased globally at the gene promoters during MSC osteogenic differentiation [47]. Another study reported that p300/CBP-associated factor (PCAF), an HAT, was required for osteogenic differentiation. PCAF, via histone acetylation of Runx2, promotes osteoblast differentiation. PCAF stimulates H3K9 acetylation at the promoters of the BMP signaling genes that control osteogenesis [48]. The Runx2/P57 P1 promoter region was accompanied by enhancement of activating histone marks, H3ac and H3K27ac, during osteogenesis of Wharton’s Jelly MSCs (WJ-MSCs) [49]. In addition to HATs, HDACs are widely involved in osteogenic differentiation of MSCs. Gordon et al. have reported downregulation of HDAC1, HDAC2, and HDAC3 during osteogenesis [50]. A study by Simic et al. showed that Sirtuin 1 (SIRT1), an HDAC, deacetylated β-catenin protein at lysine 49 and 345 (K49 and K345), which led to accumulation of β-catenin and eventually promoted osteogenesis [51].

Histone methylation is another histone modification that regulates gene expression during osteogenesis. Histone methyltransferases (HMTs) transfer up to three methyl groups to histone proteins, commonly at lysine residues, in contrast to histone demethylases (HDMs) that remove the methyl groups. These modifications activate or repress transcription depending on the location of the methylation [52]. The impact of histone methylation during MSC osteogenic differentiation has been the focus of a number of studies. Hassan et al. reported that HOXA10, a key gene for embryonic patterning of skeletal elements, was involved in differentiation of MSCs to the osteogenic lineage through activation of the Runx2, ALP, OCN, and BSP genes. They found that these effects were mediated by hyperacetylation of whole chromatin and H3K4 hypermethylation of these genes [53]. Higashihori et al. reported that H3K9 mono- and di-methyltransferase G9a was involved in osteogenesis regulation by twist gene repression [54]. HDMs have been shown to play an important role in osteo-differentiation of MSCs isolated from patients with oculo-facio-cardio-dental (OFCD) syndrome. Patients with OFCD have the BCL-6 corepressor (BCOR) mutation, which is characterized by canine teeth with long roots and craniofacial defects. MSCs obtained from these subjects had H3K4 and H3K36 hypermethylation at promoter of the AP2a gene. AP2a is an essential transcription factor for craniofacial development [55].

Zhang et al. demonstrated that JMJD3 (KDM6B), an H3K27me3 demethylase, was necessary for both intramembranous and endochondral ossification. JMJD3 might act as a co-activator of Runx2, and their cooperation promoted osteoblast differentiation and bone formation in mice [56].

H3K27me3 (repressive mark) has an important function in osteogenesis regulation. This epigenetic mark is stimulated by enhancer of zeste homology 2 (EZH2), an HMT, and is removed by lysine demethylase 6A (KDM6A). Ye et al. reported that the HDMs, KDM4B and KDM6B, regulated DLX and HOX gene expressions by removing H3K9me3 and H3K27me3, respectively, which indicated their pivotal role in osteogenic commitment of MSCs [57]. EZH2 and KDM6A also act as epigenetic switches to determine the cell fate in an osteo-adipo lineage. EZH2 and KDM6A influence the levels of H3K27me3 on the promoters of master regulatory genes. Enhanced expression of *EZH2* in MSC stimulates adipogenesis in vitro and inhibits the osteogenic differentiation potential both in vitro and in vivo, whereas KDM6A acts in the opposite manner [8]. Therefore, both histone acetylation and methylation are important in osteogenic differentiation because they control osteogenic gene expressions. The histone modification status determines the fate of MSCs; therefore, we could change the cellular fate by changing gene expressions via artificial intervention.

### Chromatin remodeling

Chromatin remodeling is the dynamic modification of chromatin by which nucleosomes move to a new location on the genomic DNA, which induces chromatin reconstruction to access DNA and control gene expression [58]. Reconstructed chromatin supplies required energy from ATP hydrolysis [59].

ATP-dependent chromatin remodelers are categorized into four distinct families: switch/sucrose-non-fermenting (SWI/SNF), imitation switch (ISWI), chromodomain-helicase-DNA-binding (CHD), and inositol requiring 80 (INO80) [60]. Other major chromatin remodeling proteins include the polycomb group (PcG) and trithorax group (TrxG) [61]. The PcG complex is involved in regulation of osteogenic differentiation of MSCs. PcG proteins form multi-protein complexes, termed polycomb-repressive complexes (PRCs). PRC2 contains EZH2, embryonic development protein (Eed), and suppressor of zeste12 (Suz12). Wei et al. investigated cyclin dependent kinase 1 (CDK1)-dependent phosphorylation of EZH2. They observed disruption of EZH2 binding with the other PRC2 components. Repression of EZH2 methyltransferase activity caused enhanced osteogenic differentiation of MSCs [62]. The role of CHD7 in osteogenesis was also supported by Yi et al. Binding of CHD7 to the enhancer region of sp7 was necessary for osteogenesis of human MSCs [59].

### MicroRNAs

miRNAs are endogenous small non-coding RNAs ~ 20 nucleotides long that post-transcriptionally regulate gene expression. miRNAs perform their actions through interaction with sites of antisense complementarity in 3′ untranslated regions (UTRs) of specific target mRNAs. They cause degradation of the target mRNAs or translational suppression depending on the degree of miRNA-mRNA complementarity [63, 64]. In humans, miRNAs target and regulate approximately more than 60% of genes responsible for protein production [65]. They contribute to the regulation of other epigenetic mechanisms such as histone acetylation and methylation [66, 67]. miRNAs act as positive or negative regulators of osteo-differentiation processes. The role of miRNAs in modulation of osteoblast differentiation of MSCs has been reported (Table 1).

**Table 1:** Various miRNAs regulating osteoblast differentiation of MSCs

miRNA	Target	Feature	Reference(s)
miR-2861	HDAC5	- Induces osteoblast differentiation	[68]
miR-433-3p	DKK1/Wnt/β-catenin pathway	- Induces osteoblast differentiation through decreasing DKK1 expression	[69]
miR-199b-5p	GSK-3b	- Promotes osteoblast differentiation	[70]
miR-15b	Smurf1	- Promotes osteoblast differentiation by indirectly protecting Runx2 protein from Smurf1-mediated degradation	[71]
miR-503	Smurf1	- Induces osteogenic differentiation via suppressing Smurf1 expression	[72]
miR-22	HDAC6	- Promotes osteogenesis and inhibits adipogenesis by repressing HDAC6	[66]
miR-20a	PPARγ, Bambi, and Crim1	- Promotes osteogenesis of hMSCs by upregulation of BMP/Runx2 signaling	[73]
miR-31, miR-93, and miR-145	Osx	- Suppresses osteogenic differentiation	[74–76]
miR-139-5p	Frizzled (FZD)	- Suppresses osteogenic differentiation	[77]
miR-154-5p	Wnt/PCP signals	- Negative regulation of ADSC osteogenic differentiation	[78]
miR-26a-5p	Wnt5a	- Suppresses osteogenic differentiation	[79]
miR-204/211	Runx2	- Inhibits osteogenesis and promotes adipogenesis of BMSCs	[80]

Collectively, the abovementioned studies illustrated that epigenetic factors regulate genes, transcription factors, and signaling pathways. Precise knowledge of epigenetic patterns in MSCs before and after differentiation is necessary to improve efficient differentiation of MSCs. These findings enable us to discover regulation of osteogenesis via epigenetic modifications. In addition, they provide clues to discover novel treatments for bone defects by targeting specific regulators involved in osteogenesis and can help us to better control the fate of MSCs in cell-based therapy and advances in regenerative medicine and tissue engineering.

## Epigenetic modifiers, known as epidrugs

The results of various experiments show that growth factors, peptides, small molecule, and epidrugs, as chemical compounds, play important roles in targeted osteogenesis. These compounds affect genes and transcription factors, and can induce MSC differentiation to osteoblasts. Some have been assessed in the in vitro studies and in vivo (animal models). However, only a few of these components have reached the clinical trial stage [81]. Table 2 lists some of the osteogenic components that induce osteogenic commitment of MSCs.

**Table 2:** Representative examples of osteoinductive components that have been investigated in osteogenesis and bone regeneration

Components	Mechanism of action	Outcome	References
**Pre-clinical studies**			
Dexamethasone	Reduction of phosphorylation of ser125	Upregulated osteogenic markers	[82]
Oxysterols	Induce the expression of the Hh target genes	Upregulated osteocalcin (OCN) and RUNX2	[83]
Purmorphamine	Activation of hedgehog signaling pathway	Upregulated RUNX2 gene during osteoblast differentiation	[84]
Simvastatins	–	Enhanced RUNX2, osterix, OCN, and COlla1	[85]
W9 (YCWSQYLCY) peptide	Activation of TGF and the PI3 kinase/Akt signaling pathway	Promote osteogenesis	[86]
IRW peptide	Activation of PI3K-Akt-RUNX2 pathway	Promote osteogenesis	[87]
GRGDS peptide	–	Promote osteoblast adhesion and proliferation	[88]
**Clinical studies**			
SP1	Reduction of osteoclast deposition on bone surfaces	Bone regeneration	[89]
BMP-2/7	**–**	Stimulate osteogenesis	[90]
Fingolimod (FTY720)	Immunomodulating drug derived from the natural product myriocin also known as fingolimod or Gilenya	Enhanced bone formation	[91]
PDGF	–	Comparable fusion rates and less pain in group with PDGF-BB treatment as compared with autograft treatment group	[92]
FGF-2	–	Enhance healing of periodontal defects	[93]
P-15	–	Significant increase in bone mineral density of bone around the implants	[94]

Epigenetics is defined as reversible heritable changes in gene expression without concomitant changes to the genomic sequence that engages in the biological process of mammalian development, and cellular differentiation and maintenance of tissue- and cell type-specific functions [95]. Extensive studies of cancers have revealed that human cancer cells harbor both genetic mutations and epigenetic aberrations [96, 97]. Accordingly, because of the pivotal reversible nature of epigenetic modifications, attention has been given to the application of epigenetic-based drugs (epidrugs). Epidrugs are drugs that target epigenetic marks, which are responsible for epigenetic alterations, such as DNMT and HDAC or mirRNAs. These drugs inhibit or activate disease-associated epigenetic proteins and lead to improvement, treatment, or prevention of diseases [95, 96]. Epidrugs, alone or with other anticancer drugs, have been used to treat cancers where epigenetic regulation has a key role [98]. For example, 5-azacitidine (Vidaza) is an FDA-approved drug for myelodysplastic syndrome (MDS) and is one of the DNMT inhibitors (DNMTi). This drug has improved the quality of life for patients with MDS [99]. 5-Aza-2′-deoxycytidine (5-Aza-dC, decitabine, Dacogen), the deoxy form of 5-azacitidine, has been used to treat ovarian cancer. It has passed a phase I clinical trial for hematopoietic malignancies [100, 101]. Dysregulation of epigenetic modifications plays a role in other conditions, including inflammation, obesity, type 2 diabetes, dyslipidemia, cardiovascular diseases, neurological disorders, and metabolic disorders [102]. Epidrugs that have the capability to improve the epigenetic imbalances in these disorders are potentially ideal candidates for future treatments.

HDACs modulate gene expression. Histone deacetylase inhibitors (HDACi) alter gene expression and are being considered as favorable drugs for the treatment of malignancy. HDACi are divided into several classes: hydroxamic acids, cyclic peptides, short-chain fatty acids, and epoxides. These inhibitors arrest cell growth and induce differentiation or apoptosis of cancer cells both in vitro and in vivo [103]. Several HDACi such as valproic acid (VPA), suberoylanilide hydroxamic acid (SAHA, vorinostat), and entinostat have been used to treat MDS, cutaneous T cell lymphoma, and breast cancer, respectively [104–106]. In addition to HDACi, the EZH2 inhibitors EPZ-6438, GSK126, GSK343, and UNC1999 have also been used in cancer therapy [96].

miRNAs are another important aspect of epigenetic regulation in cancers. The expression profiles of miRNAs deregulate during oncogenesis. Hence, Epi-miRs open up new horizons for novel cancer therapies [107]. The results of a number of studies indicate that miRNA expression is modulated by other epigenetic processes such as DNA methylation and histone modification in cancer. For example, miR-31/-34/-145/-21/-125b/-181a/-141 are downregulated in various cancers. The epigenetic drug 5-aza-2′-deoxycytidine has been used to modulate their expressions [96].

Despite promising reports on the potential application of epidrugs as therapeutic strategies, there is significant concern that surrounds the unwanted adverse and off-target effects. Hematologic complications, such as bleeding and anemia, can occur following treatment with DNMTi [108]. In mouse RMS cells, 5-Aza-dC promotes metastasis via reactivation of the pro-metastatic Ezrin gene [109]. Unfavorable adverse effects are a concern with HDACi. Most HDACi are non-selective and target multiple classes or isozymes of HDACs (pan-HDACi), and result in toxic adverse effects. The most common complaints associated with SAHA are anemia, increased blood urea, anorexia, hyperglycemia, thrombocytopenia, fatigue, and nausea. However, the toxic effects of SAHA were dose-dependent and SAHA was more efficient at a lower dose [110]. Usual adverse effects of another HDACi, VPA, included hepatic toxicity, gastrointestinal disorders, blood dyscrasias, and hyperammonemia. These adverse effects were mild, dose-dependent, and reversible. However, valproate-induced hyperammonemic encephalopathy (VHE) is a rare, concerning adverse effect [111]. Also, commonly reported adverse effects of entinostat include fatigue and gastrointestinal, hematologic, and metabolic disorders, which appear to be concentration-dependent [112]. The therapeutic approaches in the base of miRNAs as epidrugs, as well as the mentioned epidrugs, suffer from off-target problems. A single miRNA can target multiple genes and cause unwanted effects [113].

In order to reduce the unwanted adverse effects, it is necessary to precisely recognize epidrug targets in order to design selective and class or isoform-specific enzyme inhibitors for specific cells. Also, the use of a suitable drug delivery system with controlled local, sustained release and evaluation of the lowest effective dose might overcome this issue.

### Epidrugs as regulators of osteogenesis

MSCs are of tremendous interest to stem cell-based transplantation therapy; newly emerging research has implicated the important role of epigenetic mechanisms in regulating mesodermal lineage determination and differentiation. Evidence indicates that the induction of MSC differentiation toward osteoblasts is supported by epigenetic regulations [114].

There is growing evidence that pertains to the effect of epidrugs on bone formation as well as cancer clinical therapy. A number of studies have highlighted the role of DNMTi during osteogenesis. Chen et al. reported the role of 5-Aza-dC as a lineage determinator between adipogenesis and osteoblastogenesis. They found that it promotes osteoblastogenesis by demethylation and upregulation of the Wnt10a gene and osteoblastogenic markers ALP, OSX, Twist1, and Dlx5 in 3T3-L1 preadipocytes and the ST2 MSC line [115]. Lee et al. examined the effect of 5-Aza-dC on C2C12 cells. They observed that 5-Aza-dC demethylated promoters of the Dlx5 and Osx genes in a dose- and time-dependent manner. The expression levels of the ALP and OCN genes increased after 5-Aza-dC treatment [116]. Similarly, pretreatment of mouse bone marrow MSCs with 5-azacytidine increased expressions of DLX5, Runx2, COL1A1, Osx, and OCN, and accelerated osteogenesis. Hypomethylation of the DLX5 promoter occurred after pretreatment with 5-azacytidine as evidenced by the bisulfite sequencing technique [117].

In addition to DNMTi, some studies have focused on HDACi and their role during differentiation of MSCs to an osteoblast lineage. VPA is a short-chain branched fatty acid prescribed for treatment of epilepsy and neurological disorders. Cho et al. reported that treatment of human adipose tissue-derived stromal cells (hADSCs) with VPA as an HDAC inhibitor increased the expressions of OSX, OPN, Runx2, and BMP2 during osteogenesis, which indicates a positive role for VPA in osteogenesis [118]. Paino et al. assessed the impact of VPA on human dental pulp stem cells. They reported that VPA significantly enhanced matrix mineralization by enhancing OPN and bone sialoprotein (BSP) expressions, but had a negative effect on late-stage marker of differentiation (OCN), which indicated that VPA did not impact the terminal step of osteogenesis. This effect was strongly related to inhibition of HDAC2 and has implied that HDAC2 is a fundamental enzyme for osteogenesis [103]. La Noce et al. demonstrated that treatment of human dental pulp stem cells with VPA caused well-organized bone tissue formation after subcutaneous implantation of the cells into immunodeficient mice, despite a decrease in OCN expression [119].

SAHA is another HDACi that is clinically used to treat cancer. In a study, the treatment of C57BL/6J mice with SAHA caused decreased mineralization and OPN, COL1A1, and OCN gene expression, and eventually led to bone loss in rodents [120]. In contrast, other researches showed that optimum dose of SAHA had no negative effects on bone formation and could promote osteogenesis via upregulation of Runx2 and BMP-2-dependent ALP activity in both human bone marrow MSCs and an osteoporotic mice model [121, 122]. These contradicting results for SAHA in osteogenesis indicate that it has a dose-dependent function during osteo-differentiation.

Trichostatin A (TSA), a widely applied HDACi, has been assessed in bone studies. Boer et al. reported that ALP expression and bone formation were dramatically enhanced by TSA-treated human MSCs in an ex vivo cultured mouse calvaria but implantation of these cells into immune-deficient mice did not show any significant results [123]. TSA also promoted rat adipose-derived stem cell (ADSC) osteogenic differentiation by hyperacetylation of the Runx2 promoter in a BMP signaling-dependent manner [124]. Hu et al. reported that TSA potentiated BMP9-induced ALP activity, OCN, OPN, and matrix mineralization in mouse MSCs. Also, findings from a fetal limb explant culture showed the role for TSA in endochondral bone formation [125].

Sodium butyrate (NaBu) is a short-chain fatty acid that plays a critical function in the homeostasis of the gastrointestinal tract; NaBu inhibits HDACs. It has been shown that NaBu modulates osteogenic differentiation in hMSCs by an ERK-dependent Runx2 activation [126]. NaBu, by increasing H3K9ac and decreasing H3K9me onto the Runx2 promoter, has been shown to enhance rat ADSC osteogenesis [127]. Ali et al. assessed the effect of abexinostat, an HDACi, on osteoblast differentiation of human MSCs. They observed that abexinostat enhanced the expression levels of the ALP and Osx genes through the WNT signaling pathway [128].

**Fig. 2:**
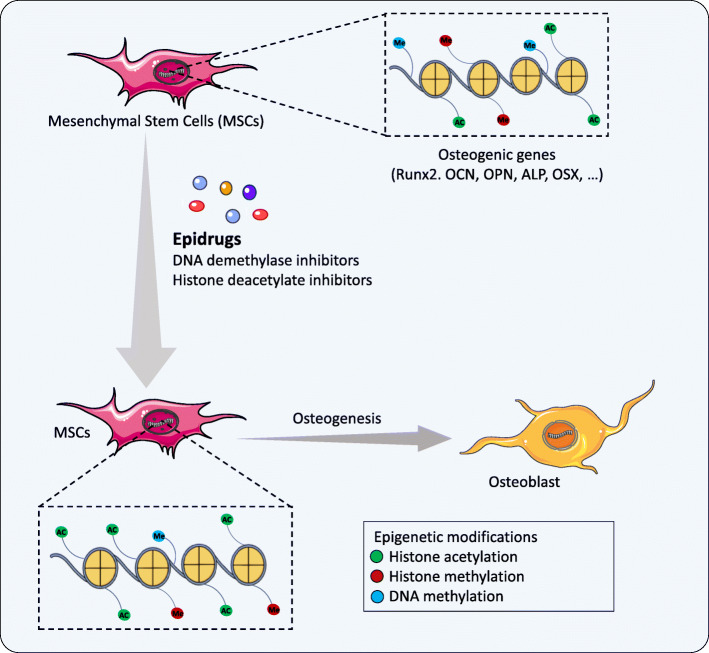
Schematic presentation of epidrugs’ effect on the promotion of osteogenesis. After treatment of MSCs with epidrugs, there is a decrease in the epigenetic marks responsible for the silencing genes (DNA methylation, histone methylation) and an increase in gene-activating epigenetic marks (histone acetylation) in osteogenic genes, which result in promotion of osteogenesis

Besides the effect of HDACi during osteoblast differentiation, several studies were conducted to assess inhibition of the EZH2 epigenetic factor, a novel therapeutic target for improvement of bone formation. Inhibition of EZH2-mediated 3-deazaneplanocin A (DZNep) promoted osteoblast differentiation of hMSCs [8]. Jing et al. sought to determine a correlation to inhibition of EZH2 via DZNep during osteoporosis. They reported that knockdown of EZH2 via DZNep decreased the level of H3K27me3 on the promoters of Wnt genes and recovered mouse osteoporotic BMSCs in vitro and in vivo [129]. GSK126, another EZH2 inhibitor, showed similar results and enhanced osteogenic commitment of human adipose-derived MSCs (hAMSCs). The results indicated that GSK126 decreased H3K27me3 levels by inhibition of EZH2, which led to promotion of ALP and Runx2 expressions [130]. More recently, Khanban et al. have reported the impact of the G9A inhibitor A366 on osteogenic potential of BMSCs. They found that Runx2, ALP, COL1A1, Osx, and OCN gene expression levels and osteogenesis decreased in BMSCs derived from A366-treated rats [131]. Table 3 lists some epidrugs and their targets involved in osteogenesis. Epidrugs can be considered to be suitable choices for targeting epigenetic aberrations in the treatment of bone defects and efficient differentiation of MSCs for application in regenerative medicine. Figure 2 shows the impact of epidrugs on the promotion of osteogenesis.

**Table 3:** Various epidrugs targeting osteogenesis

Epidrug	Alternate name	Target(s)	Impact on osteogenesis	References
5-Azacytidine	Vidaza	DNMT	Upregulation of osteogenic gene markers (ALP, Dlx5, Runx2, Col1a1, osterix, and osteocalcin)	[117]
5-Aza-2′-deoxycytidine	Decitabine	DNMT	Upregulation of Wnt10a, a key factor determining the fate of the mesenchymal lineage toward osteoblasts Increased Dlx5 and Osx genes	[115, 116]
Valproic acid	–	**HDAC**	Enhanced BMP2 expression Enhanced matrix mineralization Negative effect on OCN	[103, 118]
Vorinostat	SAHA	**HDAC**	Inhibition of immature osteoblasts **Induction of osteogenic markers (RUNX2, BMP-2, and ALP)**	[120–122]
Trichostatin A	TSA	**HDAC**	Promotion of osteogenic differentiation via increased in osteocalcin, osteopontin, and ALP	[123–125]
Sodium butyrate	**–**	**HDAC**	**Promotion of osteogenic differentiation via RUNX2**	[126, 127]
Abexinostat	–	**HDAC**	**Increased OSX and ALP**	[128]
GSK126	–	**HMT**	Acceleration of osteogenic differentiation by regulation of Bglap, Sparc, Spp1, and Ibsp genes	[130]
3-Deazaneplanocin A	DZNep	**HMT**	Upregulation of Wnt1, Wnt6, and Wnt10a osteogenic genes	[129]

The survival of MSCs is a significant point to be made about the use of epidrugs in promotion of MSC differentiation. The results of studies have shown that the viability of MSCs was affected by various doses of epidrugs; increased doses had a negative impact on MSC survival [117, 119, 132]. An evaluation of the effect of VPA and NaBu on MSCs showed a dose-dependent reduction in MSC proliferation. In addition, the proliferation of MSCs from diverse origins (umbilical cord blood, adipose tissue, and bone marrow) has different sensitivities to epidrugs [133]. In a recent study, Salami et al. have reported that, in addition to the concentration of VPA, the medium context could have a key role in the effects of VPA on Wharton’s Jelly MSCs [134]. These findings highlight the importance of determining a suitable dose of epidrugs with minimal cytotoxicity in different cells and contexts before their use in vitro and in vivo.

## Future trends and concluding remarks

MSC characteristics include their ability to be isolated from various sources, multipotent properties, immunosuppressive capacity, no risk of tumorigenesis, and easy expansion in vitro. These characteristics make them ideal candidates for regenerative medicine and tissue engineering. However, the use of these cells for stem cell-based therapy still requires a rigorous understanding of the mechanisms that occur during in vitro and in vivo MSC differentiation. Osteogenesis is controlled by numerous genes, transcription factors, signaling pathways, and epigenetic mechanisms. Nevertheless, detailed knowledge of epigenetic regulation of osteoblast differentiation is obscure. Hence, a novel insight into prevention and treatment of disease-associated epigenetic defects such as cancer would include the identification and targeting of epigenetic modification enzymes by epidrugs. Recently, the use of epidrugs to treat various cancers has been studied. Some have been approved by the FDA, and several are in phase I/II studies. The role of some epigenetic enzyme inhibitors has been addressed in osteoblast differentiation of MSCs, and the results have shown that some of these epidrugs improve osteogenesis and bone formation via alterations in epigenetic arrangements. According to the emerging knowledge on the role of these epigenetic modifiers in osteogenesis, epidrugs can be potentially used to improve in vitro and in vivo osteogenic differentiation with the goal to enhance the capability of MSCs to be used in tissue engineering and regenerative medicine. Epidrugs have the ability to be used as treatment for metabolic bone disorders such as osteoarthritis and osteoporosis; however, cytotoxicity is the main problem for their clinical use. In order to overcome the adverse effects of an epidrug, the first step would be to identify which epigenetic marker should be targeted in a certain disease and use its class or isoform-specific enzyme inhibitors. Furthermore, additional research is needed to determine the optimal dose and discover the appropriate epidrug transfer systems prior to their clinical applications in bone tissue engineering. Currently, a gap exists between our knowledge and clinical applications, and a more thorough investigation in this context would be essential prior to the use of epidrugs as therapy. It is, therefore, necessary that further research should be conducted to fully understand the epigenetic changes that occur with bone disorders and to recognize epigenetic patterns in genes responsible for their repair and rejuvenation.

## Data Availability

Data sharing is not applicable to this article as no datasets were generated or analyzed during the current study.
